# Uncovering T cell-specific differential expression patterns associated with pollen exposure in individuals with allergic rhinitis

**DOI:** 10.1186/1710-1492-10-S2-A66

**Published:** 2014-12-18

**Authors:** Chen Xi Yang, Casey P Shannon, Amrit Singh, Anne K Ellis, Scott J Tebbutt

**Affiliations:** 1 UBC James Hogg Research Centre and Centre for Heart + Lung Innovation, University of British Columbia, Vancouver, British Columbia, V6Z 1Y6, Canada; 2 Kingston General Hospital and Queen’s University, Kingston, Ontario, K7L 2V7, Canada; 3 Prevention of Organ Failure (PROOF) Centre of Excellence, Vancouver, British Columbia, V6Z 2K5, Canada

## Background

Investigating transcriptomics in whole blood is a promising avenue of research for helping to understand the allergic response [1]. However, the heterogeneity of peripheral whole blood significantly complicates the interpretation of whole blood expression data. Statistical deconvolution approaches, which can model and infer the sample composition and the cell type-specific expression, may provide a powerful means of studying complex tissues, such as whole blood, in an integrated fashion [2].

## Methods

14 individuals with allergic rhinitis were simultaneously exposed to ragweed pollen in the Environmental Exposure Unit. Peripheral blood samples were collected using PAXgene Blood RNA tubes before and after the 3 hours of pollen exposure. Gene expression profiling was performed using Affymetrix GeneChip® Human Gene 1.0 ST Arrays. Publicly available expression data (E-GEOD-48558) was used to estimate the cellular composition from whole blood expression profiles. The estimated proportions were compared against those measured by an automated hematology analyzer.

## Results

The estimated proportions of lymphocytes, granulocytes and monocytes were compared against the observed proportions. The prediction was good in lymphocytes (R^2^=0.70, RMSE=0.036) and granulocytes (R^2^=0.75, RMSE= 0.046), but relatively poor in monocytes (R^2^=0.52, RMSE=0.030). No significant changes in cellular proportions between pre-challenge and post-challenge samples were identified. 261 (110 up-regulated and 151 down-regulated) differentially expressed probe sets were identified comparing pre and post-challenge samples at a false discovery rate (FDR) of 10%.

## Conclusions

Statistical deconvolution is accurate in predicting the cellular proportions of lymphocytes and granulocytes but relatively poor in predicting monocytes. Allergen exposure causes significant changes in the blood transcriptomes of participants with allergic rhinitis undergoing pollen exposure. The inferred proportions will be used for cell type-specific significance analysis of microarrays (csSAM), to assess differential expression in T cells. The CD4^+^ T cell expression profiles from E-GEOD-43497, a similar study of allergic rhinitis, will be used to validate our findings.
